# A Novel Melanocortin-4 Receptor Mutation MC4R-P272L Associated with Severe Obesity Has Increased Propensity To Be Ubiquitinated in the ER in the Face of Correct Folding

**DOI:** 10.1371/journal.pone.0050894

**Published:** 2012-12-12

**Authors:** Susana Granell, Clara Serra-Juhé, Gabriel Á. Martos-Moreno, Francisca Díaz, Luis A. Pérez-Jurado, Giulia Baldini, Jesús Argente

**Affiliations:** 1 Department of Biochemistry and Molecular Biology, University of Arkansas for Medical Sciences, Little Rock, Arkansas, United States of America; 2 Unitat de Genètica, Universitat Pompeu Fabra, and Centro de Investigación Biomédica en Red de Enfermedades Raras (CIBERER), Instituto de Salud Carlos III, Barcelona, Spain; 3 Department of Pediatric Endocrinology, Hospital Infantil Universitario Niño Jesús, Instituto de Investigación La Princesa, Madrid, Spain; 4 Department of Pediatrics, Universidad Autónoma de Madrid and Centro de Investigación Biomédica en Red de fisiopatología de la obesidad y nutrición (CIBERobn), Instituto de Salud Carlos III, Madrid, Spain; University of Córdoba, Spain

## Abstract

Heterozygous mutations in the melanocortin-4 receptor (*MC4R*) gene represent the most frequent cause of monogenic obesity in humans. *MC4R* mutation analysis in a cohort of 77 children with morbid obesity identified previously unreported heterozygous mutations (P272L, N74I) in two patients inherited from their obese mothers. A rare polymorphism (I251L, allelic frequency: 1/100) reported to protect against obesity was found in another obese patient. When expressed in neuronal cells, the cell surface abundance of wild-type MC4R and of the N74I and I251L variants and the cAMP generated by these receptors in response to exposure to the agonist, α-MSH, were not different. Conversely, MC4R P272L was retained in the endoplasmic reticulum and had reduced cell surface expression and signaling (by ≈3-fold). The chemical chaperone PBA, which promotes protein folding of wild-type MC4R, had minimal effects on the distribution and signaling of the P272L variant. In contrast, incubation with UBE-41, a specific inhibitor of ubiquitin activating enzyme E1, inhibited ubiquitination of MC4R P272L and increased its cell surface expression and signaling to similar levels as wild-type MC4R. UBE41 had much less profound effects on MC4R I316S, another obesity-linked MC4R variant trapped in the ER. These data suggest that P272L is retained in the ER by a propensity to be ubiquitinated in the face of correct folding, which is only minimally shared by MC4R I316S. Thus, studies that combine clinical screening of obese patients and investigation of the functional defects of the obesity-linked *MC4R* variants can identify specific ways to correct these defects and are the first steps towards personalized medicine.

## Introduction

As the incidence of obesity in children and adolescents has risen dramatically in occidental countries [1], numerous studies have focused on understanding its cause. This has led to the identification of different forms of monogenic obesity, with mutations in the melanocortin-4 receptor (*MC4R*) gene, a single exon gene on 18q22, being the most frequent [2]. MC4R is a G-protein coupled receptor (GPCR) expressed in the brain where its activation is involved in the control of food intake and energy expenditure [3]–[6]. Fasting induces the expression of orexigenic neuropeptides including Agouti-related peptide (AgRP) [7], while fed conditions stimulate the expression of proopeiomelanocortin (POMC), the precursor to alpha-melanocyte stimulating hormone (α-MSH) [8]. The balance between the effects of these two neuropeptides on MC4R activation is critical to control food intake, energy expenditure and body weight [3]–[6]. Neurons producing α-MSH project to neurons in the hypothalamic paraventricular nucleus, where this neuropeptide binds to MC4R leading to activation of adenylate cyclase-dependent production of cAMP. Another population of neurons located in the hypothalamic arcuate nucleus synthesize the MC4R antagonist AgRP [3], [7], [9]. Activation of MC4R leads to suppression of food intake and food seeking behavior [3]–[6], emphasizing the importance of this system in controlling body weight.

The first cases of obesity due to *MC4R* mutations were reported in 1998 [10], [11], with the prevalence of mutations in this gene now estimated at 1/1000 and accounting for up to 6% of severe obesity cases [2], [12]. Since then, more than 150 different *MC4R* variants have been described [2], [13]–[15]. Carriers of obesity-linked *MC4R* variants present increased body mass index (BMI), hyperphagia, and hyperinsulinemia [2]. Characterization of *MC4R* mutations in *in vitro* cell systems has resulted in the identification of different mechanisms by which obesity-linked *MC4R* variants can cause defective function [16]. In approximately 85% of the cases studied to date the cell surface expression of the receptor is decreased, with MC4R being retained in an intracellular location [13], [17]–[19]. We have recently reported that obesity-linked *MC4R* variants are misfolded in the endoplasmic reticulum (ER) and targeted for degradation by ER-associated degradation (ERAD) [20]. In addition, some of the obesity-linked *MC4R* variants have defective binding affinities and/or signaling responses to endogenous or synthetic agonists [13], [21]. Here we present a study where 77 children with early onset morbid obesity were screened for mutations in *MC4R*. We found 2 new variants, P272L and N74I, in *MC4R*. The cellular localization, signalling properties upon agonist stimulation and possible pharmacological rescue of these new mutations are reported.

## Patients and Methods

### Ethics Statement

This study was approved by the Ethics Committee for Clinical Investigation at the Hospital Infantil Universitario Niño Jesús (Madrid, Spain) with the protocol number R-0014/10 and written informed consent was given by parents or guardians on behalf of the patients.

### Patients

To be included in the study the patient had to be prepubertal and obese (defined as BMI>3 SDS for the Spanish population according to their chronological age and sex), with no signs of any syndromic abnormality or other disease. The obesity had to be of early onset (before 3 yrs of age). A total of 77 white children (50 males and 27 females) were invited to participate.

### Mutation analysis

DNA from the proband and first degree relatives was extracted from peripheral lymphocytes using a Qiagen Midi Kit (Qiagen, Hilden, Germany). Control DNA samples from 200 healthy white donors were from the National DNA Bank (Salamanca, Spain). The *MC4R* gene was PCR-amplified and Sanger-sequenced (primers and conditions available upon request). To assess the putative outcome of the detected non-synonymous single nucleotide changes, we used the Condel tool that provides a consensus deleteriousness score based on the combination of various tools (http://bg.upf.edu/condel/about; [22]).

### Reagents and Antibodies

Lipofectamine 2000 and the mouse monoclonal anti-FLAG antibodies were purchased from Invitrogen (Carslbad, CA). Rat monoclonal anti-hemaglutinin (HA) antibody (3F10), peroxidase (POD)-conjugated anti-HA antibody, POD-conjugated anti-mouse IgG, protease inhibitor (Complete Mini), and 2,2′-azino-bis(3-ethylbenzthiazoline-6-sulfonic acid (ABTS) tablets were from Roche Applied Science (Indianapolis, IN); α-MSH, 3-isobutyl-1-methylxanthine (IBMX) and 4-phenyl butyric acid (PBA) were from Sigma-Aldrich (St. Louis, MO); POD-conjugated anti-mouse IgG was from Pierce Biotechnology, Inc (Rockford, IL); Cy3-conjugated anti-rat and Cy3-conjugated anti-rabbit IgG antibodies were from Jackson ImmunoResearch (West Grove, PA). Rabbit polyclonal anti-calnexin, mouse monoclonal anti-KDEL and the cAMP ELISA Kit were from Enzo Life Sciences (San Diego, CA). The enhanced chemiluminescence detection kits were from PerkinElmer Life Sciences, Inc (Boston, MA). The ubiquitin activating enzyme inhibitor (UBE1–41) was from Biogenova (Maryland, USA) [23]. Neuroblastoma Neuro2A (N2A) cells were a kind gift from Peter Cserjesi, (Tulane University, New Orleans, LA).

### Constructs

The WT HA-MC4R-GFP plasmid was described earlier [24]. The primers used to generate HA-MC4R-GFP (N74I, P272L and I251L) mutants were 5′GGCAATAGCCAAGAACAAGATCCTGCATTCACCCATG3′, 5′TTCTACATCTCTTGTCTTCAGAATCCATATTGTGTGTGC3′ and 5′ACCTTGACCATCCTGCTTGGCGTCTTTGTTGTC3′. All mutations were confirmed by sequencing. Plasmid DNA for transfections into mammalian cells was isolated using the DNA purification system from Promega. Flag-ubiquitin was a kind gift from Dr. JoAnn Trejo, University of North Carolina at Chapel Hill.

### Cell culture, transfection and drug treatment

N2A cells (American Type Culture Collection, Manassas, VA, USA) were cultured in DMEM with 10% fetal bovine serum and penicillin/streptomycin. Cells were transiently transfected using lipofectamine 2000 by following the manufacturer's instructions. Experiments were carried out 48 h after transfection unless indicated otherwise. For drug treatment, vehicle alone (DMSO) or 2 mM PBA was added 16 h after transfection and maintained until the cells were harvested. For the inhibition of ubiquitination 20 µM UBE1–41 was added 16 h before the cells were harvested.

### Gel electrophoresis and immunoblotting

Separation of proteins by SDS-PAGE and immunoblotting with the indicated antibodies were performed as described [20]. Unless noted otherwise, cell lysates were prepared by scraping cells from 60 mm diameter plates in 0.4 ml of sample buffer containing protease inhibitors. Samples were sonicated 3 times for 2 s periods and loaded onto SDS-PAGE.

### Immunoprecipitation

Transfected cells grown in 60 mm dishes were scraped in immunoprecipitation (IP) buffer (100 mM Tris-HCl, pH 7.4, 50 mM NaCl, 1% Triton, and protease inhibitors) and incubated for 30 min at 4°C. Cell lysates were rotated for 1 h at 4°C with the indicated antibody. After addition of protein A/G beads, the samples were further incubated for 1 h at 4°C. After three washes with IP buffer the beads were resuspended in sample buffer, boiled for 5 min, and centrifuged. Supernatants and cell extracts were loaded onto a SDS-PAGE gel.

### Fluorescence microscopy

Epifluorescent images were captured with a CARV I spinning confocal imaging system (BioVision Technologies, Exton, PA) attached to an X-71 fluorescence microscope (Olympus, Tokyo, Japan). Images were collected using a CoolSNAP HQ camera (Photometrics, Tucson, AZ). Confocal images were captured using an Olympus FluoView™ FV1000 confocal microscope and analyzed by using ImageJ software version 1.33 by Wayne Rasband (NIH, Bethesda, Maryland).

### Quantification of MC4R expressed at the cell surface

Cells were washed three times with DMEM and incubated in DMEM for 1 h at 37°C, DMEM was aspirated, cells were transferred on ice and incubated in the same medium with rat monoclonal anti-HA antibodies for 1 h at 4°C. Cells were then fixed with 4% formaldehyde, washed with PBS, and incubated with PBS containing 100 µg/ml ovalbumin and Cy3-conjugated anti-rat antibodies without addition of detergents. Cells expressing HA-MC4R-GFP were chosen at random and the Region of Interest (ROI) was drawn on the merged image around the compartment labeled by the HA antibodies using ImageJ software. The amount of HA-MC4R-GFP at the cell surface was estimated by measuring the fluorescence intensity of the receptor in the Cy3 channel.

### Quantification of HA-MC4R-GFP at the cell surface by enzyme-linked immunoassay

HA-MC4R-GFP at the cell surface by enzyme-linked assay was determined as described previously [24]. Briefly, N2A cells transiently transfected as indicated were washed three times with DMEM and incubated in DMEM for 1 h at 37°C. Cells were further incubated for 1 h at 4°C in the same medium in the presence of POD-conjugated anti-HA antibodies (25 mU/ml). Cells were washed 2 times with ice-cold DMEM and fixed with formaldehyde at 4°C for 10 min. Cells were washed 3 times with PBS and POD activity was determined by using the ABTS substrate to measure the amount of receptor at the cell surface.

### Assay to determine cAMP

Cells were washed with DMEM and incubated with the same medium containing 0.5 mM IBMX for 10 min and then stimulated with 100 nM α-MSH for 15 min at 37°C. The medium was aspirated and intracellular cAMP was measured by using the immunoassay kit by Enzo life technologies following the manufacturer's instructions.

### Statistical Analysis

Data are expressed as mean ± S.D. GraphPad Prism version 5.0 (GraphPad software, San Diego, CA) was used to perform unpaired t-test or one-way analysis of variance (ANOVA). Statistical significance was chosen as p<0.05.

## Results

### Two novel *MC4R* mutations, P272L and N74I, identified in obese patients

Two novel heterozygous mutations were identified in unrelated patients from this cohort of 77 cases with early onset severe obesity (2.6%), as well as a rare polymorphism in a third case.

**Figure 1:**
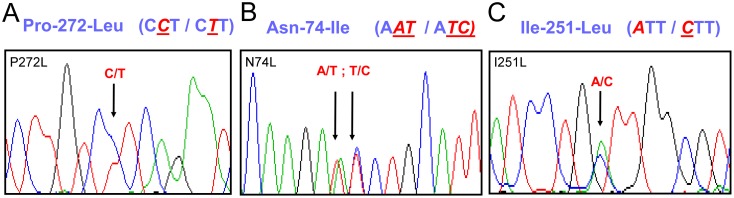
Chomatograms of the three identified *MC4R* mutations. Chromatograms of *MC4R* P272L (A), *MC4R* N74L (B) and *MC4R* I251L (C).

Case #1: A white male had a novel c.815C>T (p.P272L) mutation in *MC4R* (Fig. 1A) inherited from his mother who also had early-onset obesity. The patient was born at 33 weeks of gestation with a birth weight of +3.2 SDS and birth length in the 85^th^ percentile. At presentation he was 6^10/12^ yrs old with a BMI of +5.6 SDS.

Case #2: A male of Arabic ancestry carried a novel c.221_222AT>TC (p.N74I) *MC4R* mutation (Fig. 1B) inherited from his obese mother. His birth weight was +2.09 SDS at 40 weeks of gestation (length unknown). At presentation he was 2^7/12^ yrs old with a BMI of +4.8 SDS.

Case #3: A previously described *MC4R* polymorphism (rs52820871, p.I251L; Fig. 1C) was identified in a female that was obese from the first months of live. She was 10 yrs old at clinical evaluation with a BMI of +3.7 (SDS).

The P272L and N74I mutations have not been described in the SNP database (www.ncbi.nih.org/projects/SNP) nor in 6,503 controls (13,006 chromosomes) included in the exome-variant-server database [25]. In addition, none of the 200 healthy individuals from Spain tested carried either mutation. To assess the putative outcome of the detected non-synonymous single nucleotide changes, we used the Condel method [22] that provides a consensus deleteriousness score based on the combination of various tools. The scores of different methods (SIFT, Polyphen2 and MutationAssessor) are weighted using the complementary cumulative distributions produced by the five methods on a dataset of approximately 20000 missense SNPs, both deleterious and neutral. The probability that a predicted deleterious mutation is not a false positive of the method and the probability that a predicted neutral mutation is not a false negative are employed as weights (http://bg.upf.edu/condel/about; [22]). Any Condel score above 0.5 is considered as likely deleterious, with the maximun score being 1. By this analysis, integrating protein structure and interaction predictions with conservation information, both mutations are predicted to be highly deleterious, with N74I having a Condel score of 1 and P272L a score of 0.99. These mutations are located in the 1^st^ intracytoplasmatic loop and the 3^rd^ extracellular loop, respectively. In contrast, the I251L variant, located in the 6^th^ transmembrane domain, is found in ∼0.9% of chromosomes and predicted to be functionally neutral with a Condel score of 0.

### P272L-*MC4R* variant is retained in the ER

**Figure 2:**
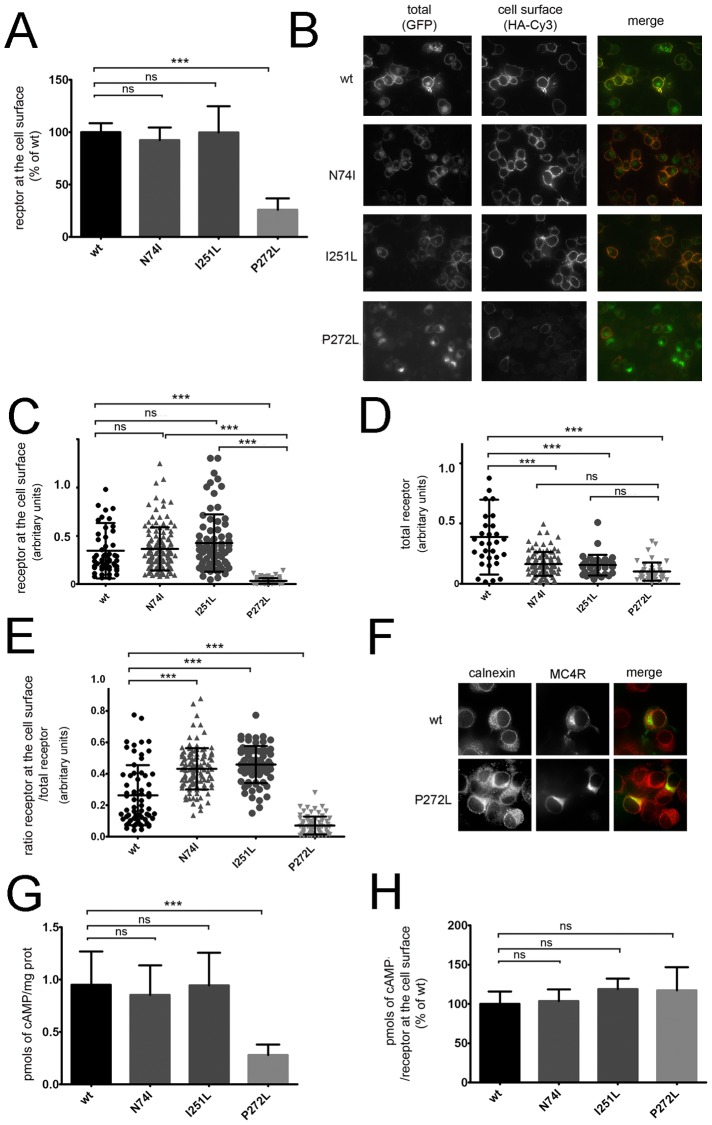
Cellular localization of the distinct *MC4R* mutations. **A**) N2A cells were transfected with wt-HA-MC4R-GFP and mutated HA-MC4R-GFP (N74L, I251L and P272L) or incubated at 37°C for 48 h. Receptor at the cell surface was measured by enzyme-linked immunoassay. Data are expressed as percentage of the wt receptor, (n = 3). **B**) N2A cells were transiently transfected as in A. Cells were transferred at 4°C, incubated with primary rat monoclonal anti-HA antibodies, fixed, and incubated with secondary Cy3-conjugated anti-rat antibodies. **C–E**) Quantification of the confocal images of the experiment shown in B. The ratio of HA-MC4R-GFP at the cell surface/total receptor in the cell (E) was estimated by dividing the fluorescence intensity of the receptor at the cell surface (Cy3, C) by the fluorescence intensity of the receptor in the entire cell (GFP, D). wt-HA-MC4R-GFP (n = 64) and mutated HA-MC4R-GFP (N74L n = 111, I251L n = 74 and P272L n = 72). Statistical significance, *** = p<0.0001. **F**) N2A cells were transiently transfected with wt-HA-MC4R-GFP and mutated HA-MC4R-GFP P272L and incubated at 37°C for 48 h. Cells were fixed, incubated with primary rabbit polyclonal anti-calnexin antibodies (Enzo Life Science, Farmingdale, NY) and with secondary Cy3-conjugated anti-rabbit antibodies. **G–H**) N2A cells were transiently transfected with wt-HA-MC4R-GFP and mutated HA-MC4R-GFP (N74L, I251L or P272L) and incubated at 37°C for 48 h. Cells were stimulated with 100 nm α-MSH for 15 min at 37°C. Intracellular cAMP was measured by using the immunoassay kit by Assay Designs (San Diego, CA), following the manufacturer's instructions. The amount of cAMP generated was expressed as pmols cAMP/mg prot (G), and pmols cAMP/receptor at the cell surface (H), (n = 3). ).

Mutated HA-MC4R-GFP P272L expression at the cell surface was decreased compared to the wt-receptor (26.05±10.81%, Fig. 2A), suggesting that this variant is retained in an intracellular compartment. HA-MC4R-GFP N74I and I251L were expressed at the cell surface at levels similar to wt. Cell surface expression of N74I- and I251L-HA-MC4R-GFP, measured by immunofluorescence, was similar to wt, while that of HA-MC4R-GFP P272L was decreased (Fig. 2B and 2C), which is consistent with the POD-based immunoassay data (Fig. 2A). All mutants had reduced total MC4R expression compared to wt, as estimated by GFP fluorescence, with the greatest effect observed in the P272L mutant (Fig. 2D). The ratio of Cy3 fluorescence (receptor at the plasma membrane)/GFP fluorescence (total receptor) indicates the fraction of total receptor at the plasma membrane. This ratio was decreased by approximately 3-fold for the obesity-associated HA-MC4R-GFP P272L mutant as compared to wt, indicating intracellular retention similar to other obesity-associated MC4R mutants that are misfolded in the ER (Fig. 2E) [20]. Conversely, the fraction of N74I and I251L receptor at the cell surface was increased. Thus, the variants have different cellular distribution, with HA-MC4R-GFP P272L being retained in the ER with increased colocalization with the ER chaperone calnexin (Fig. 2F) and with HA-MC4R-GFP N74I and I251L having a reduced total pool of receptors, but an increased fraction of receptor at the plasma membrane.

The amount of intracellular cAMP per mg of cell protein produced after α-MSH stimulation was unchanged in N2A cells expressing HA-MC4R-GFP N74I or I251L and reduced by approximately 70% in the P272L mutation compared to wt (Fig. 2G). The amount of cAMP produced per receptor at the cell surface was unaffected (Fig. 2H), suggesting that HA-MC4R-GFP P272L, N74I and I251L do not have defective signalling.

### Inherent propensity to be ubiquitinated, rather than misfolded, retains MC4R P272L in the ER

**Figure 3:**
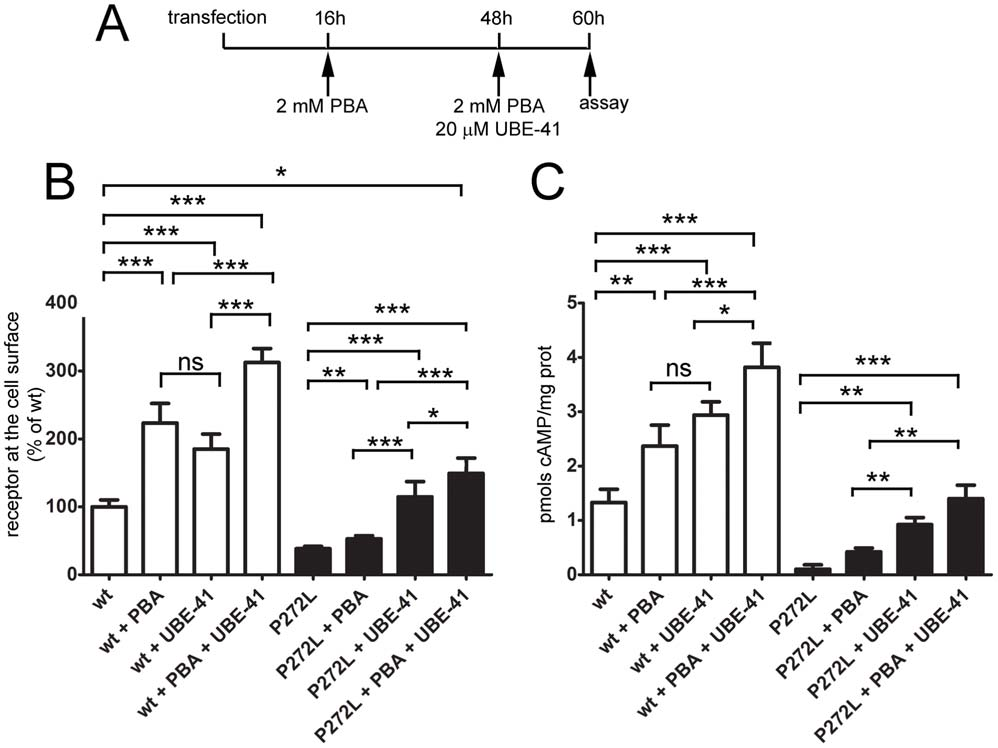
Functional studies and rescue of the mutation P272L. **A**) N2A cells were transiently transfected with wt-HA-MC4R-GFP and mutated HA-MC4R-GFP-P272L and treated with vehicle or 2 mM PBA, 20 µM UBE-41 or both at the time points indicated in the diagram. **B**) Receptor at the cell surface from cells in A was measured by a spectrophotometric assay. Data are expressed as percentage of the wt without treatment, (n = 3). **C**) Intracellular cAMP from cells in A was measured by using the immunoassay kit by Assay Designs (San Diego, CA), following the manufacturer's instructions. The amount of cAMP generated by was expressed as pmols cAMP/mg protein, (n = 3). Statistical significance, * = p<0.05,** = p<0.001,*** = p<0.0001, ns = p>0.05.

We have previously shown that cell exposure to a chemical chaperone, PBA, increases plasma membrane expression of wt-MC4R and of some obesity-associated MC4R variants that are retained in the ER [20]. UBE1-41, a specific inhibitor of ubiquitin-activating enzyme 1 [23], also improved cell surface expression of wt-MC4R and of the obesity-associated MC4R I316S variant [20]. The effects of PBA and UBE1-41 were additive, suggesting that the two drugs affect MC4R exit from the ER by targeting steps that are, at least in part, independent of each other. Here, in a similar experimental approach (Fig. 3A), we found that UBE-41 promoted not only increased expression of wt-HA-MC4R-GFP at the cell surface (Fig. 3B), but also increased agonist-dependent generation of cAMP (Fig. 3C). Therefore, it appears that a drug that specifically inhibits ubiquitination can, by itself, promote exit of functional MC4R from the ER. These experiments suggest that although a population of wt-MC4R can fold correctly it nevertheless has the propensity to be ubiquitinated, and that this feature contributes to its retention in the ER. The effects induced by PBA on cell surface expression and signaling of wt-HA-MC4R-GFP were similar in magnitude to those induced by UBE1-41 (by ∼2-fold, Fig. 3B). Conversely, exposure to UBE-41 increased cell surface expression and signaling by HA-MC4R-GFP P272L to a much higher extent than that observed after exposure to PBA (by ∼1.4-fold for PBA and by ∼3-fold for UBE-41, Fig. 3B). Thus, in the case of MC4R P272L, as compared to wt-MC4R, most of HA-MC4R-GFP P272L retained in the ER is because of the receptor's tendency to be ubiquitinated, rather than to misfold.

### The mechanism by which MC4R P272L and MC4R I316S are retained in the ER is different

**Figure 4:**
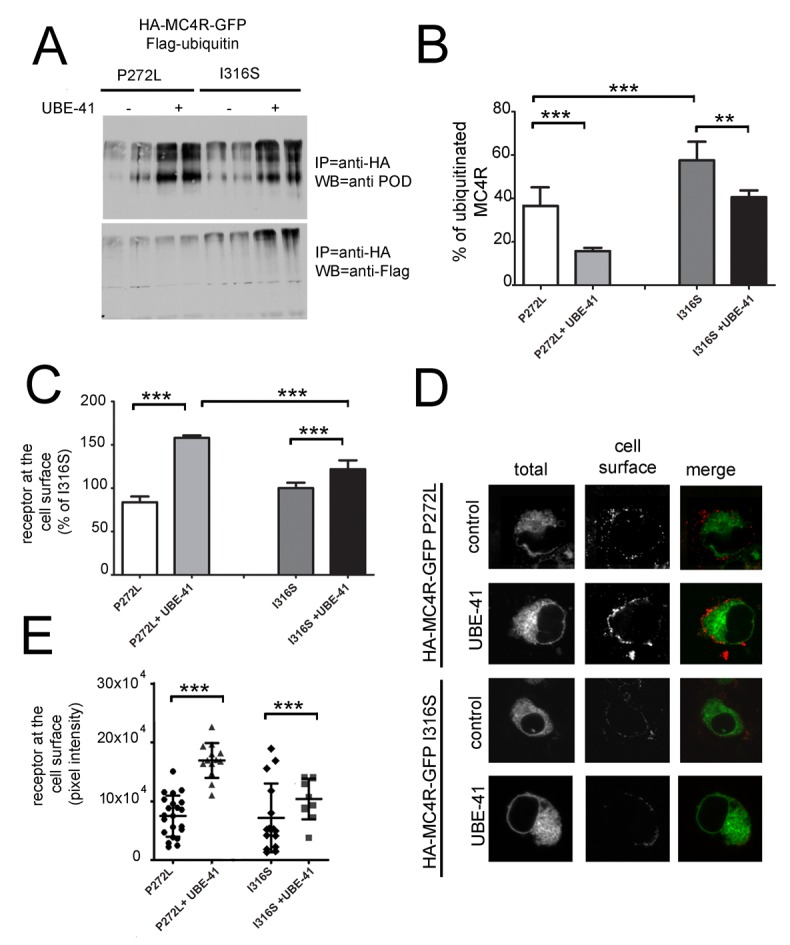
Functional studies comparing different *MC4R* mutations. **A**) N2A cells were transiently co-transfected with mutated HA-MC4R-GFP P272L or HA-MC4R-GFP I316S and FLAG-ubiquitin. Immunoprecipitation (IP) from cell lysates was carried out with anti-HA antibodies and analyzed by Western blot (WB) by using the indicated antibodies. **B**) The graph shows the quantification of the experiment shown in A. The data are expressed as % of ubiquitinated MC4R obtained from the ratio anti-FLAG immunoreactivity/anti-HA immunoreactivity, (n = 3, including that shown in A). **C**) N2A cells were transiently transfected with mutated HA-MC4R-GFP P272L or HA-MC4R-GFP I316S and treated with vehicle or 20 µM UBE-41 for 16 h. Receptor at the cell surface was measured by a spectrophotometric assay. Data are expressed as percentage of the obesity-linked MC4R I316S variant without treatment, (n = 3). **D**) N2A cells were transiently transfected and treated as in A. Cells were transferred at 4°C, incubated with primary rat monoclonal anti-HA antibodies, fixed, and incubated with secondary Cy3-conjugated anti-rat antibodies. **E**) The graph shows the quantification of the experiment shown in D. (P272L n = 22, P272L+UBE-41 n = 13, n = 15 and I316S+I316S+UBE-41 = 10). **D**) Statistical significance, ** = p<0.001,*** = p<0.0001.

The results shown in Figure 3 demonstrate that functional MC4R P272L can be rescued to the cell surface more efficiently by UBE-41 than by PBA exposure. This is different than what was observed for another obesity-linked variant retained in the ER, MC4R I316S, which was rescued to the cell surface similarly by UBE-41 and by PBA exposure [20]. This posed the question as to whether obesity-linked receptor variants differ in the main mechanism by which they are retained in the ER, with MC4R I316S having more propensity to misfold and then to be ubiquitinated and with MC4R P272L being ubiquitinated in the face of correct folding. We reasoned that a different response to UBE-41 exposure might help discriminate differences in the mechanism by which MC4R P272L and MC4R I316S are retained in the ER. To monitor the extent by which HA-MC4R-GFP P272L and I316S are ubiquitinated, the receptors were co-expressed with FLAG-ubiquitin. The amount of HA-MC4R-GFP in the cell with and without UBE-41 treatment was measured by Western blot analysis of the immunoprecipitated receptor and the extent of its ubiquitination by the associated Flag immunoreactivity. In the absence of UBE-41, the percentage of ubiquitinated HA-MC4R-GFP P272L was less than HA-MC4R-GFP I316S (∼37% and 60% respectively, Figs. 4A and 4B). After exposure to UBE1-4, the total amount of HA-MC4R-GFP P272L and I316S was increased similarly, by approximately 5-fold (Fig. 4A). This indicates that inhibition of ubiquitination leads to decreased disposal of MC4R both in the case of the P272L and of the I316S variants. However, the fraction of ubiquitinated HA-MC4R-GFP was decreased by ∼70% in the case of the P272L variant and by ∼25% in the case of *MC4R* I316S variant (Figs. 4A and 4B). This experiment shows that I316S can still be ubiquitinated under conditions where most of P272L is not. Importantly, treatment with UBE-41 improved cell surface expression of MC4R P272L by more than 70%, measured both by immunoassay (Fig. 4C) and by immunofluorescence (Figs. 4D and 4E). Conversely, MC4R I316S expression at the cell surface was only increased by ∼25% by the treatment with the inhibitor (Figs. 4C–E). These data indicate that under conditions where the ubiquitination capacity of the cell is decreased, the propensity of MC4R P272L to be ubiquitinated is also reduced and traffic of the receptor outside the ER to the cell surface is restored. This is different from MC4R I316S, which, under the same conditions, remains mostly ubiquitinated and localized to the ER.

## Discussion

We describe two novel heterozygous mutations in the *MC4R* gene associated with early onset morbid obesity in childhood. These mutations, P272L and N74I, are predicted to be highly deleterious and cosegregate with obesity in the respective kindred. In contrast, the I251L variant is found in almost 1% of chromosomes and predicted to be functionally neutral.

When studied in an *in vitro* system, the P272L mutation was retained in the ER failing to reach the plasma membrane, which resulted in reduced signaling after agonist stimulation. Thus, retention in the ER and poor expression of the *MC4R* P272L mutant at the cell surface could explain the obese phenotype observed in patients carrying this mutation. In contrast, we cannot explain the pathogenic mechanism of the N74I and I251L *MC4R* variants, if any, as expression at the plasma membrane and intracellular cAMP generation appeared to be normal after heterologous transfection of the mutant constructs. However, the N74I described here resulted in a reduction in the total number of MC4R/cell suggesting a deleterious effect. The pathogenic mechanism of this mutation might depend on the interaction of the mutant with the wild-type protein in heterozygous individuals and thus be missed by our approach. Whether the affinity of the receptor for one or both of its endogenous ligands, AgRP and α-MSH, is affected by these mutations remains to be determined. Furthermore, as expression of this receptor on presynaptic terminals is also important for metabolic control [26], it is possible that trafficking of the N74I mutated receptor to other cellular locations is affected. Likewise, MC4R has been recently shown to be expressed in astrocytes [27]. Given the recent interest in astrocyte involvement in metabolic control [28], modifications in its expression and functions in this cell type could also possibly be involved in producing an obese phenotype.

We have recently reported that some obesity-linked MC4R variants are retained in the ER because they are misfolded [20]. In the same study, we found that the chemical chaperone PBA can increase folding and plasma membrane expression of wt-*MC4R* and of some obesity-linked *MC4R* variants including I316S, with increased signaling after stimulation with the agonist. In contrast, here we show that the *MC4R* P272L variant can be rescued to the cell surface more efficiently by decreasing its ubiquitination than by treating with PBA to increase ER folding capacity. These results suggest that most of *MC4R* P272L is retained in the ER due to an intrinsic tendency to be ubiquitinated, rather than misfolded and then ubiquitinated. In this respect, *MC4R* P272L rescued at the cell surface by cell incubation with UBE-41 is functional, demonstrated by an increase of signaling after agonist stimulation that parallels its increase at the cell surface. The results presented here suggest that strategies aimed to decrease ubiquitination of some obesity-linked variants could be a main target for novel therapeutic approaches.

UBE-41 inhibits the ubiquitin activating enzyme (E1) [23], which catalyzes the first step on the ubiquitination reaction and is not substrate-specific. In contrast, the ubiquitin protein ligases (E3), which catalyze the last step of the ubiquitination process, are substrate-specific and therefore represent a more suitable therapeutic target [29]. Whether silencing of E3 is able to increase surface expression and function of obesity-linked MC4R variants and whether modulation of the activity of E3 *in vivo* could protect against the obesity associated with mutations in MC4R are topics to be addressed in future studies.

The frequency of *MC4R* gene mutations found in the population of obese children reported here (1.4–2.6%) is lower than in other studies, 5.8% [2], [12]–[19]. This could be related to the strict selection of patients, including only those with extreme obesity very early in life. Thus, mutations causing a less severe or delayed form of obesity were not included. The polymorphism I251L associated with obesity in the patient described here is reported to protect against obesity [21], [30]–[32]. This variant increased the fraction of MC4R localized at the plasma membrane, but decreased overall abundance of the receptor. While it is possible that the obesity presented by this subject is not related to this polymorphism, further studies are necessary to understand the intracellular trafficking and function of the I251L variant as the protective effects of this polymorphism are surely influenced by other genetic factors.

In conclusion, the results reported here emphasize the importance of functional studies in order to determine the mechanisms involved in monogenic obesity, with these functional studies possibly paving the way for the development of personalized medical treatment of specific forms of obesity.
